# *INSIG2 *gene polymorphism is associated with increased subcutaneous fat in women and poor response to resistance training in men

**DOI:** 10.1186/1471-2350-9-117

**Published:** 2008-12-23

**Authors:** Funda E Orkunoglu-Suer, Heather Gordish-Dressman, Priscilla M Clarkson, Paul D Thompson, Theodore J Angelopoulos, Paul M Gordon, Niall M Moyna, Linda S Pescatello, Paul S Visich, Robert F Zoeller, Brennan Harmon, Richard L Seip, Eric P Hoffman, Joseph M Devaney

**Affiliations:** 1 Research Center for Genetic Medicine, Children's National Medical Center, Washington DC, 20010, USA; 2 Department of Exercise Science, University of Massachusetts, Amherst, MA 01003, USA; 3 Division of Cardiology, Henry Low Heart Center, Hartford Hospital, Hartford, CT 06102, USA; 4 Center for Lifestyle Medicine and Department of Health Professions, University of Central Florida, Orlando, FL 32816, USA; 5 Department of Physical Medicine and Rehabilitation, School of Medicine, University of Michigan, Ann Arbor, MI 48108, USA; 6 Department of Sport Science and Health, Dublin City University, Dublin 9, Ireland, UK; 7 Department of Kinesiology, University of Connecticut, Storrs, CT 06269, USA; 8 Human Performance Laboratory, Central Michigan University, Mount Pleasant, MI 48859, USA; 9 Department of Exercise Science and Health Promotion, Florida Atlantic University, Davie, FL 33314, USA

## Abstract

**Background:**

A common SNP upstream of the INSIG2 gene, rs7566605 (g.-10,1025G>C, Chr2:118,552,255, NT_022135.15), was reported to be associated with obesity (Body Mass Index, [BMI]) in a genome-wide association scan using the Framingham Heart Study but has not been reproduced in other cohorts. As BMI is a relatively insensitive measure of adiposity that is subject to many confounding variables, we sought to determine the relationship between the INSIG2 SNP and subcutaneous fat volumes measured by MRI in a young adult population.

**Methods:**

We genotyped the INSIG2 SNP rs7566605 in college-aged population enrolled in a controlled resistance-training program, (the Functional Polymorphism Associated with Human Muscle Size and Strength, FAMuSS cohort, n = 752 volunteers 18–40 yrs). In this longitudinal study, we examined the effect of the INSIG2 polymorphism on subcutaneous fat and muscle volumes of the upper arm measured by magnetic resonance imaging (MRI) before and after 12 wks of resistance training. Gene/phenotype associations were tested using an analysis of covariance model with age and weight as covariates. Further, the % variation in each phenotype attributable to genotype was determined using hierarchical models and tested with a likelihood ratio test.

**Results:**

Women with a copy of the C allele had higher levels of baseline subcutaneous fat (GG: n = 139; 243473 ± 5713 mm^3 ^vs. GC/CC: n = 181; 268521 ± 5003 mm^3^; p = 0.0011); but men did not show any such association. Men homozygous for the G ancestral allele showed a loss of subcutaneous fat, while those with one or two copies of the C allele gained a greater percentage of subcutaneous fat with resistance training (GG: n = 103; 1.02% ± 1.74% vs. GC/CC: n = 93; 6.39% ± 1.82%; p = 0.035).

**Conclusion:**

Our results show that the *INSIG2 *rs7566605 polymorphism underlies variation in subcutaneous adiposity in young adult women and suppresses the positive effects of resistance training on men. This supports and extends the original finding that there is an association between measures of obesity and *INSIG2 *rs7566605 and further implicates this polymorphism in fat regulation.

## Background

Obesity is increasing in prevalence and is an important public health problem. In the United States, 17.1% of children 2 to 19 years are overweight and 32.2% of adults aged > 20 years are obese. The prevalence of being overweight among children and adolescents and obesity among men increased significantly between 1999 and 2004 [1]. Obesity is a major risk factor for the development of hypertension, diabetes, coronary heart disease, and stroke [1]. Genetic predisposition is known to underlie much of the obese and morbid obesity phenotype, with approximately 50% of effect environmental (diet, exercise) and between 30–70% genetically determined [2]. Phenotypic measures of obesity vary considerably between different studies, and this can complicate the interpretation of genetic association studies. Body mass index (BMI) is a frequently used measure of obesity, yet it considers only weight as a function of height, and does not measure fat distribution (visceral vs. subcutaneous) or relative contribution of muscle and fat to the BMI. As a result, professional body builders are often classified as obese or morbidly obese by measures of BMI [2,3].

Herbert et al. [4] conducted a genome-wide association study to identify genetic loci (single nucleotide polymorphisms; SNPs) contributing to obesity using BMI as the primary phenotype. The authors reported strong statistical evidence that the *INSIG2 *promoter SNP rs7566605 (g.-10,1025G>C, Chr2:118,552,255, NT_022135.15) was associated with increased BMI in a cohort of the 694 participants in the Framingham Heart Study [4]. In this initial report, association of rs7566605 with BMI was replicated in ethnically distinct populations, including European and African Americans. *INSIG2 *is a ~21.5 Kb gene located on chromosome 2q14 that has been functionally linked to lipid metabolism, most notably due to its role in endogenous cholesterol and fatty acid synthesis feedback inhibition [5]. It is an endoplasmic reticulum membrane bound protein that inhibits the proteolytic activation of Sterol Response Element Binding Proteins (SREPs) in response to cholesterol or insulin [5]. Animal data suggest a role for *INSIG2 *in regulating triglyceride levels in rats [6].

Data mapping of quantitative trait loci in mice (QTL) for obesity related traits and their response to high fat diet data have shown linkage to the *INSIG2 *gene region and with obesity related traits such as fat depots (reproductive, renal, mesenteric, and inguinal) and serum cholesterol levels [7].

A series of follow-up studies sought to validate the association of *INSIG2 *rs7566605 with markers of obesity in humans, with ten publications reporting inconsistent findings [8-18]. Considering the larger studies, Loos et al. [14] used the British European Prospective Investigation of Cancer (EPIC) Norfolk study, an older population (mean age 58 when DNA sample collected), and did not find a significant association with BMI. Similarly, Dina et al. [9] French Données Epidémiologiques sur le Syndrome d'Insulino-Résistance (DESIR) also studied older insulin resistant subjects, and did not find an association with BMI. In addition, the results from the Nurses Health Study (NHS), that consists of women age 47, did not replicate the original study by Herbert *et al*. [4]. The analysis of German participants from the SHIP study by Rosskopf *et al*. [16] did not report a positive association with BMI when the samples were stratified by genotype and gender; however, the subgroup of obese samples (BMI > 30) have a higher frequency of the C allele than individuals that are over-weight (BMI: 25–30). Interestingly, a recent study examined the effect of genotype on weight loss (one year intervention) and found that children with the CC genotype lost less weight than children with the GC/GG genotype [15]. Many of the non-replications of the reported associated polymorphism and obesity may result from a lack of power given the low incidence of obesity in many of the populations examined.

Most of the previous studies on *INSIG2 *rs766605 have been performed on patient cohorts or relatives of probands recruited because of end stage disease phenotypes such as hypertension, heart disease, cancer, type 2 diabetes mellitus and have used the relatively non-sensitive and non-specific phenotype of BMI as a measure for obesity [8-18]. We sought to use a more specific and sensitive measure of adiposity in younger adults (average age 24 yrs), namely volumetric MRI measures of subcutaneous fat of the upper arm [19-22], to test for associations of *INSIG2 *genotype and subcutaneous fat. We also opted to stratify for gender, and limit to Caucasians, given variability seen in previous associations of this SNP. Finally, we examined if *INSIG2 *genotype affected change in subcutaneous fat with resistance training.

## Methods

### Subjects

The FAMuSS cohort is described elsewhere [21]. Briefly, the population was derived from a multicenter, NIH funded study designed to identify genetic factors that dictate baseline bone, muscle and fat volume and the variability in response to exercise training. 752 healthy Caucasian volunteers (18–40 yrs, FMS cohort; 451 women; 301 men; see Table 1) were enrolled in this study by one of the 8 centers (University of Massachusetts Amherst, University of Connecticut, Dublin University (Ireland), Florida Atlantic University, Hartford Hospital, University of Central Florida, West Virginia University, Central Michigan University). The study was approved by the Children's National Medical Center Institutional Review Board (protocol #2449), and was in compliance with the Helsinki Declaration. Participants were asked to maintain their typical food intake, physical activity and drug use (e.g. oral contraceptives) during the 12 wk period.

**Table 1:** Subject Characteristics for the FAMuSS study

Characteristic	Women		Men	
	**N**	**Mean ± SD**	**N**	**Mean ± SD**

Age (years)	320	22.87 ± 5.34	197	23.89 ± 5.71

Baseline weight (kg)	320	64.71 ± 12.44	197	78.77 ± 14.54

Baseline height (cm)	320	164.23 ± 6.73	197	178.46 ± 6.64

Baseline BMI	320	23.68 ± 4.28	197	24.68 ± 4.06

Baseline subcutaneous fat (trained arm)	320	257641 ± 116264	197	172577 ± 90778

Baseline subcutaneous fat (untrained arm)	319*	259102 ± 118598	197	176227 ± 97396

% change in subcutaneous fat (trained arm)	320	6.44 ± 67.05	197	3.56 ± 17.70

% change in subcutaneous fat (untrained arm)	319*	1.80 ± 10.42	197	1.02 ± 17.50

* One female had an unreadable MRI image of the untrained arm.

### Exercise Training Program

Resistant training (RT) was performed with the non-dominant arm (trained arm). The protocol was described elsewhere [21]. Briefly it consisted of two 45 – 60 minute sessions per week for 12 weeks. Each session was supervised by an exercise physiologist professional or a trained student. Before each session, participants warmed-up with 2 sets of 12 repetitions of the biceps preacher curl and the seated overhead triceps extension. Each session included dumbbell biceps curls, dumbbell biceps preacher curls, and incline dumbbell biceps curls, overhead dumbbell triceps extension, and dumbbell triceps kickbacks. The amount of weight was aggressively increased during the 12 wks.

### MRI measurement

In the FAMuSS study, we were only able to obtain useable MRI images from 517 of the individuals enrolled due to the quality of some of the scans (see Table 1). Entry MRI was done 24 to 48 h before the isometric or 1RM test. Scans post-training MRI was performed 48 – 96 h after the last training session. Fifteen 16 mm contiguous axial slices from each arm were taken from each arm independently. The top of the bead in a sagittal scout view was used to locate the 8^th ^slice going from the top of the arm toward the elbow. Scans for both arms were taken by Fast Spoiled Gradient Recalled (FSPPGR) and Fast Spin Echo (FSE) with TE 1.8/TR 200 msec. All 8 centers submitted the MRI data to the Research Center for Genetic Medicine at Children's National Medical Center (CNMC) in Washington, D.C, via e-mail or DAT disc, and all scans were integrated into the study SQL database.

For both cross-sectional and volumetric analysis of the MRI images, we used Rapidia (INFINITT Inc, Seoul Korea), a PC based software that allows the semi-automatic quantification of muscle, bone and subcutaneous fat [21,23]. Volume measures were taken using an anatomical landmark (metaphyseal-diaphyseal junction of the humerus) as our starting point and assayed the six 1 cM slices proximal to it. Measurement values were automatically written and saved in a SQL database together with anthropomorphic and genotyping data.

### Genotyping

Blood samples were obtained from all individuals in EDTA anticoagulant, sent to the coordinating site in Washington, DC, without subject identification. The DNA was isolated using Puregene kits (Gentra Systems, MN). Genotyping was done using a TaqMan allele discrimination assay that employs the 5' nuclease activity of Taq polymerase to detect a fluorescent reporter signal (Applied Biosystems, assayID: C_29404113_20). Both alleles were detected simultaneously using allele-specific oligonucleotides labeled with different fluorophores, and genotypes determined by the ratio of the two fluorophores used. The PCR for the each SNP contained 20 ng of DNA, 900 nM primers, 200 nM probes, and TaqMan Universal PCR Master Mix, No AmpErase UNG Applied Biosystems, Foster City, CA) in a final volume of 15 μL. PCR was performed on an MJ Research Tetrad thermal cycler (Waltham, MA). The PCR profile was 10 minutes at 95°C (denaturation), 44 cycles of 15 seconds at 92°C, and 1 minute at an annealing temperature of 60°C. Reactions were set up using a MWG robot, and fluorescence ratios and allele calling done using an ABI 7900. To test for reproducibility of our genotyping assay, 96 DNA samples were tested three times independently, and genotypes compared. There was 100% concordance of genotypes in all three replicates, with no dropouts, no calls, or ambiguous calls.

### Statistical analysis

All analyses were stratified by gender as this is a well known factor in genotype-phenotype correlations in anthropomorphic measures, such as adiposity. Hardy-Weinberg equilibrium was tested using a Chi-square analysis with one degree of freedom. Statistical tests of normality (Shapiro-Francia normality test) showed minor deviations from normality, but visual inspection of phenotype histograms showed normal distributions. This and the relatively high n's for our sample led us to use parametric tests. Associations between the SNP and each phenotype were testing using ANCOVA. Bivarate correlations between phenotype and body weight and age showed strong relationships; therefore each model included baseline body weight and age as covariates. The % variability attributable to genotype was determined by comparing the full model (containing genotype and covariates) with the constrained model (containing covariates only). Likelihood-ratio tests were done to determine if the variability attributable to genotype was statistically significant. Analyses used a nominal p-value of 0.05 as significant. To address to some extent the problem of multiple testing, we compared our unadjusted p-values to a significance level of 0.007 (Bonferroni-adjusted for 7 phenotypes tested). All analyses used Stata V10 (StataCorp, College Station, TX).

## Results

In our population of individuals that are of European descent (Whites), the G allele frequency was 0.69 and C allele was 0.31 (GG: n = 350, GC: n = 331, CC: n = 71). This matches well with the allele frequencies from the HAPMAP data set that reports an allele frequency for the G allele of 0.725 and a frequency of 0.275 for the C allele in the thirty U.S. trios provided samples, which were collected in 1980 from U.S. residents with northern and western European ancestry by the Centre d'Etude du Polymorphisme Humain. The rs7566605 variant was in Hardy Weinberg Equilibrium in our population (p = 0.3).

For subcutaneous fat in the upper trained arm (Table 2), women had baseline volumes of 257641 ± 116264 mm^3 ^and gained an average of 5900 ± 31141 mm^3 ^(2.2%) of subcutaneous fat after the 12 weeks exercise intervention. Similar values were obtained for both trained and untrained arms, indicating a systemic effect of exercise on subcutaneous adiposity. The untrained arm showed a mean baseline volume of 2601186 ± 120065 mm^3 ^with a gain of 3668 ± 29388 mm^3 ^(1.4%) that was not significantly different from the trained arm. As expected, men showed less baseline subcutaneous fat than women (172577 ± 90778 mm^3^). In addition, men gained 2.5% subcutaneous fat volume (4368 ± 24211 mm^3^) with single arm resistance training after the 12 wk intervention. The untrained arm showed a mean baseline volume of 176227 ± 97396 mm^3 ^with a gain of 312 ± 29076 mm^3 ^(0.2%) that was not significantly different from the trained arm.

**Table 2:** Baseline and percent (%) fat and strength change in FAMUSS cohort with resistance training

Phenotype	Arm	Females (N = 320) mean ± SD	Males (N = 197) mean ± SD
Baseline subcutaneous fat volume (mm^3^)	Trained	257641 ± 116264	172577 ± 90778

	Untrained	260186 ± 120065*	176227 ± 97396

Subcutaneous fat volume gained with training (mm^3^)	Trained	5900 ± 31141	4368 ± 24211

	Untrained	3668 ± 29388*	312 ± 29076

* N = 319 due to one missing MRI phenotype in females

**Figure 1:**
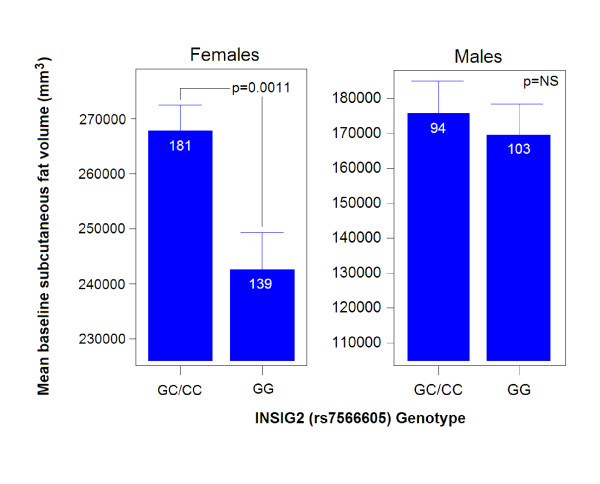
**Significant association of *INSIG2 *rs7566605 genotype with baseline subcutaneous fat volume in white men and women**. In women, a copy of the C allele was associated with more subcutaneous fat. No significant differences were seen for subcutaneous fat volume in men. This model included baseline body weight and age as covariates.

The *INSIG2 *SNP was associated with baseline subcutaneous fat volume (Table 3) (n = 320) in women in both arms (trained arm: GG: n = 139; 243473 ± 5713 mm^3 ^vs. GC/CC: n = 181; 268521 ± 5003 mm^3 ^[p = 0.001] and untrained arm: GG: n = 138; 246095 ± 5682 mm^3 ^vs. GC/CC: n = 180; 270940 ± 4978 mm^3 ^[p = 0.001]). Women that were homozygous for the G allele had 9.3% less subcutaneous fat than women in the trained and untrained arm with a copy of the C allele (Figure 1). This SNP accounted for 1.1% of genotypic variance for subcutaneous fat in women.

**Table 3:** Associations with *INSIG2 *rs7566605 phenotypes with baseline and percent (%) fat and strength change in FAMUSS cohort with resistance training.

Phenotype	Gender	P-value	N; mean ± SEM	95% CI	% variability attributable to genotype; LRT p-value
Baseline subcutaneous fat volume (mm^3^)	Female	0.0011	GG (N = 139; 243473 ± 5713) GC/CC (N = 181; 268521 ± 5003)	GG (232233 – 254714) GC/CC (258677 – 278365)	1.1%; 0.0010

Baseline subcutaneous fat volume (mm^3^)	Male	0.3557	GG (N = 103; 176503 ± 6100) GC/CC (N = 94; 168275 ± 6389)	GG (164472 – 188534) GC/CC (155675 – 180876)	0.2%; 0.3501

% change in subcutaneous fat volume	Female	0.2683	GG (N = 139; 11.20 ± 5.71) GC/CC (N = 181; 2.78 ± 4.99)	GG (-0.02 – 22.43) GC/CC (-7.05 – 12.61)	0.4%; 0.2649

% change in subcutaneous fat volume	Male	0.0353	GG (N = 103; 1.00 ± 1.74) GC/CC (N = 94; 6.39 ± 1.82)	GG (-2.43 – 4.43) GC/CC (2.78 – 10.01)	2.3%; 0.0332

All genetic models were dominant.

Men with two copies of the G allele gained less percent subcutaneous fat in the exercised arm than men with a copy of the C allele (GG: n = 103; 1.02% ± 1.74% vs. GC/CC: n = 93; 6.39% ± 1.82%; p = 0.035) (Table 3). Men with a copy of the C allele gained 5.4% more subcutaneous fat than those men with two copies of the G allele. There was no significant change in subcutaneous fat in the untrained arm (GG: n = 103; 0.9% ± 1.7% vs. GC/CC: n = 93; 1.2% ± 1.8%; p=NS) (data not shown). Interestingly, men with a copy of the C allele and a BMI < 30, gained 6.5% more subcutaneous fat (GG: n = 97; 0.79 ± 1.84 vs. GC/CC: n = 81; 7.31 ± 2.02; p = 0.019). BMI was assessed as a continuous phenotype with rs7566605 using ANCOVA models (age as only covariate) in gender specific cohorts. We did not find a statistically significant association between BMI and rs7566605 (p= 0.35 for females and p= 0.32 for males) in our study. Men with a BMI ≥ 30 did not show significant gains in fat with a copy of the C allele; however, the numbers were very small (n = 18). There was also no significant changes in subcutaneous fat in the untrained arm for BMI stratified men (BMI < 30: GG: 0.8% ± 1.8% vs. GC/CC: 1.1% ± 2.0% and BMI ≥ 30: GG: 1.2% ± 4.0 vs. GC/CC: 2.1% ± 2.8). Women did not show an association with percent changes in subcutaneous fat with resistance training.

Other phenotypes tested for association with the *INSIG2 *SNP and the p-values are located in additional table 1. Adjustment for multiple testing (7 phenotypes in male and female cohorts) showed only the baseline subcutaneous fat in females to remain statistically significant.

## Discussion

The associations of *INSIG2 *polymorphisms with approximate measures of adiposity (BMI) have varied from cohort to cohort (see additional table 2). Although BMI is widely used as a surrogate measure of adiposity, it is a measure of excess weight, rather than excess body fat, relative to height [2,3,20]. A study of BMI in more than 400 college athletes and non-athletes [24] reported that in most cases the student's BMI did not accurately reflect his or her percentage of body fat. We used a highly specific and sensitive measure of adiposity, namely volumetric semi-automated MRI volumes of upper arm, before and after a unilateral 12 wk resistance training intervention. This allowed us to assess the association of rs7566605 with baseline values of fat in the upper arm and also the effect of the polymorphism with regards to loss or gain of fat volumes following an intervention. It is important to note that most previous studies of *INSIG2 *have focused on older populations, with other morbidities, whereas we focused on a young adult healthy volunteer population. Therefore, our population was not exposed to as many environmental influences as the older populations previously studied making our genetic association data less affected by cofounding variables. Subjects were instructed to maintain activity levels, food intake, and drug use at similar levels before and during the intervention. A post-hoc analysis using oral contraceptive/hormone as a covariate in females showed no significant effect. Calorie intake and activity levels outside the strength intervention were not specifically quantified, and this is a potential limitation to this study.

We found the rs7566605 C allele to be associated with increased baseline levels of subcutaneous fat in women but not men (Figure 1). Our findings with baseline subcutaneous fat in young adult women are in general agreement with studies that have found the C allele to be associated with increased adiposity by other measures (BMI). Lyon et al. genotyped rs7566605 in across multiple ethnicities (total N = 16,969) and observed a significant (*p *< 0.05) association with BMI in 5 cohorts out 8. The authors suggested that the effect of SNP rs7566605 on BMI may be heterogeneous across population samples. However, in the same paper, a deCode cohort (N = 5,187) showed a strong negative association between BMI and rs7566605. Our findings are in general agreement with Lyons et al. where the C allele in young adult females is associated with increased adiposity as measured by subcutaneous fat volumes of the arm. In our young adult cohort, we did not show a significant association between BMI and rs7566605 for either sex (p = 0.35 for females and p = 0.32 for males). Subcutaneous fat volumes are thought to be a better measure of adiposity in young populations, while BMI becomes a better phenotype in older adults (see text below). Previous studies of associations of rs7566605 used BMI, studied older cohorts, and generally did not stratify by gender.

The recent articles have suggested there appear to be significant gender differences in the storage and utilization of fats [25]. Compared to males females have more subcutaneous adipose tissue and intramuscular triacylglycerides. Furthermore, there is higher lipoprotein lipase activity in adipocytes in males and this is correlated with estrogen concentrations [25]. During endurance exercise there is greater fat oxidation for females as compared to males, and this may direct to glycogen and protein sparing [25]. This may be related to greater insulin sensitivity in females mediating an increase in free fatty acid synthase and may also change with INSIG2 genotypes.

Particularly interesting is our finding of the associations of rs7566605 C allele with loss and gain of subcutaneous fat and muscle strength after a unilateral resistance training of the arm; however these associations were not significant if adjusted for multiple testing. Men generally gained subcutaneous fat volume in both trained and untrained arms (2–3% gain), but men with one or more copies of the C allele showed 6–7% gains (Table 3). These data suggest that exercise training may indeed lead to poorer health outcomes in *INSIG2 *C carriers, in that even limited localized exercise may trigger an increase in adiposity. This finding is similar to a study of *PPARα *L162V polymorphism in this same population, where male carriers of the rare allele also gained considerably greater adiposity following unilateral resistance training of one arm [22]. For the *INSIG2 *polymorphism, the increased subcutaneous fat gain in men was exacerbated by an unexpected decrease in muscle strength by isometric measures. Our findings agree with a previous study that examined rs7566605 in 293 obese children (mean age 10.8 years, 45% male, mean BMI 28.1 kg/m^2^) who participated in a 1-year exercise intervention [15]. Children with the CC genotype lost less weight after the intervention than children with a copy of the G allele (p = 0.007). This variant plays a role in weight regulation that needs to be explored further.

In our study, we did not analyze visceral adipose tissue (VAT) but measured the subcutaneous fat in the upper arm. Multiple studies have pointed out that VAT is more closely associated with metabolic syndrome markers [26], and that VAT secretes adipocytokines and other vasoactive substances that directly influences the risk of developing metabolic traits [27]. However, many of these associations have been done on older subjects, and visceral fat generally increases with age, while subcutaneous fat may decrease (particularly in aged subjects). There are also numerous studies that show that both subcutaneous and VAT fat can be associated with diabetes [27] and insulin resistance [28,29]. We feel that our study may provide a particularly sensitive window on adiposity, as our volumetric MRI done at a relatively young age should not be subject to many of the uncontrolled confounding factors active in previous studies.

## Conclusion

The findings reported here show that a part of inter-individual response to resistance training can be explained by *INSIG2 *genotype. We need further studies to pinpoint the effect that *INSIG2 *genotype has on the response of fat to exercise and our exercise study did not control for diet. However, this may be one of the first studies to examine the effect of a genotype on the response of fat to exercise and with this data we may begin to unravel genetics behind the differences in fat response to exercise.

## Competing interests

The authors declare that they have no competing interests.

## Authors' contributions

FS designed the study, designed genotyping assay, genotyped families, analyzed data, and drafted the manuscript. HD did statistics analyzed and interpreted data and drafted the manuscript. PMC, PDT, TJA, PMG, NMM, LSP, PSV, RFZ, RLS collected the subjects, and the clinical data, supervised exercise trainings. BH assisted genotyping, analyzing data. JMD and EPH designed the study, collected samples, and drafted the manuscript. All authors contributed to the final critical revision of the manuscript.

## Pre-publication history

The pre-publication history for this paper can be accessed here:



## Supplementary Material

Additional Table 1 **Associations of *INSIG2 *rs7566605 in the FAMUSS cohort. **The data provided represents the statistical analysis of associations with rs7566605 in FAMUSS cohort. Click here for file

Additional Table 2 **Summary Table of Results for the *INSIG2 *variant in the literature up to March 01 2008.** This worksheet summarized and compares previous study results on INSIG2 rs7566605. Click here for file
