# Supplementary material for: INSIG2 gene polymorphism is associated with increased subcutaneous fat in women and poor response to resistance training in men
Source: BMC Med Genet. 2008 Dec 23;9:117. doi: 10.1186/1471-2350-9-117 (PMC2646703; doi:10.1186/1471-2350-9-117)
Supplement: Additional Table 1 — Associations of INSIG2 rs7566605 in the FAMUSS cohort. The data provided represents the statistical analysis of associations with rs7566605 in FAMUSS cohort. [file 1471-2350-9-117-S1.doc]

| **Phenotype** | **Gender** | **p-value** | **N; adjusted means ± SEM** | **95% CI** |
| --- | --- | --- | --- | --- |
| Baseline whole arm volume | Female | 0.0081 | GG (N=139; 527681 ± 5743)  GC/CC (N=181; 548030 ± 5016) | GG (516382 – 538981)  GC/CC (536160 – 557899) |
| Difference in whole arm volume | Female | 0.8127 | GG (N=139; 44666 ± 3384)  GC/CC (N=181; 43598 ± 2956) | GG (38007 – 51324)  GC/CC (37782 – 49414) |
| Baseline whole muscle volume | Female | 0.6131 | GG (N=139; 458253 ± 10672)  GC/CC (N=181; 465449 ± 9346) | GG (437256 – 479250)  GC/CC (447060 – 483839) |
| Difference in whole muscle volume | Female | 0.8728 | GG (N=139; 43884 ± 3111)  GC/CC (N=181; 43220 ± 2725) | GG (N37763 – 50006)  GC/CC (37859 ± 48581) |
| Baseline whole arm volume | Male | 0.8037 | GG (N=103; 617431 ± 6678)  GC/CC (N=94;615010 ± 6995) | GG (604259 – 630603)  GC/CC (601214 – 628805) |
| Difference in whole arm volume | Male | 0.2821 | GG (N=103; 72550 ± 4383)  GC/CC (N=94; 79456 ± 4615) | GG (63905 – 81195)  GC/CC (70353 – 88559) |
| Baseline whole muscle volume | Male | 0.6995 | GG (N=103; 560278 ± 9354)  GC/CC (N=94; 555011 ± 9797) | GG (541829 – 578728)  GC/CC (535688 – 574334) |
| Difference in whole muscle volume | Male | 0.2778 | GG (N=103; 72361 ± 4344)  GC/CC (N=94; 79268 ± 4575) | GG (63792 – 80930)  GC/CC (70245 – 88292) |
